# Optic neuritis: A South African hospital-based prospective study protocol

**DOI:** 10.1371/journal.pone.0269514

**Published:** 2022-06-10

**Authors:** Naseer Ally, Hassan Dawood Alli, Trishal Jeeva-Patel, Andre Mochan, Eitzaz Sadiq, Ismail Mayet, Marianne Kuenast, Leisha Rajkumar

**Affiliations:** 1 Division of Ophthalmology, Department of Neurosciences, University of the Witwatersrand, Johannesburg, South Africa; 2 Division of Neurology, Department of Neurosciences, University of the Witwatersrand, Johannesburg, South Africa; 3 Department of Radiology, University of the Witwatersrand, Johannesburg, South Africa; Save Sight Institute, AUSTRALIA

## Abstract

**Background:**

Optic neuritis is a relatively common disease with an estimated lifetime risk of 0.6 per 1000; the estimated prevalence is 1–5 per 100 000/year. It occurs because of inflammation of the optic nerve from a variety of causes. The diagnosis of the disorder is established clinically and current literature is predominantly based on white patients from high-income countries. Optic neuritis presents differently in black patients compared to white patients. This study aims to assess the presentation and outcome of optic neuritis patients in a South African setting.

**Methods:**

This is a prospective, hospital-based cohort study that will enrol patients with optic neuritis presenting to either the neurology department at Chris Hani Baragwanath Academic Hospital or the ophthalmology department at St John Eye Hospital, both in Johannesburg, South Africa. The specific aetiologies, clinical presentation and radiological findings will be studied, and the patient’s course over one year will be documented in three-monthly follow-up visits. A specific group of patients with Neuromyelitis Optica Spectrum Disorders (NMOSD) and Myelin Oligodendrocyte Glycoprotein Associated Disorders (MOGAD) optic neuritis will be followed up for 5 years at yearly intervals.

**Discussion:**

This study represents one of the few cohort studies in Sub-Saharan Africa that seeks to investigate optic neuritis. Our hope is that it will lead to better insights regarding the presentation, course and outcome of this condition. We will also analyse the data with a view of developing a predictive model for good visual outcome.

## 1. Introduction

Optic neuritis (ON) is a relatively common disease with an estimated lifetime risk of 0.6 per 1000; the estimated prevalence is 1–5 per 100 000/year [1, 2]. The global annual incidence of unilateral ON is approximately 0.94–2.18 per 100 000 [3]. It occurs because of inflammation of the optic nerve from a variety of causes. It is characterised by acute visual loss that progressively worsens over a few days [3–5]. The diagnosis is established clinically and it may present to a variety of medical practitioners including neurologists and ophthalmologists [6]. While African American patients were previously thought to have a lower incidence of Multiple Sclerosis [7], the findings of more recent studies suggest otherwise [8–10]. A South African retrospective study by Pokroy et al., has shown that black patients present differently compared to white patients [11].

Optic neuritis can be classified both by clinical criteria and by aetiology. It can be divided, clinically, into typical and atypical forms [6, 12] Aetiologically, ON can be divided into Multiple Sclerosis-related ON, Neuromyelitis Optica Spectrum of Disorders-related ON (NMOSD-ON), Myelin Oligodendrocyte Glycoprotein Associated Disorders (MOGAD), ON related to systemic diseases (e.g. Sarcoidosis and Syphilis) and isolated ON not related to any systemic disease [3]. The distinction between aetiologies is important because it guides the investigations and management [13].

Typical optic neuritis (T-ON) refers to optic neuritis associated with Multiple Sclerosis (MS) which commonly occurs in the white population in regions of high MS prevalence [3, 6]. Clinical features of T-ON include a unilateral presentation with pain, especially with ocular movement, that precedes loss of vision which spontaneously recovers after 10 to 15 days. The loss of vision generally occurs over a few days and although usually mild, can range from blurring of vision to no perception of light. The presence of a relative afferent pupillary defect (RAPD) is a key clinical feature of ON. Patients may also commonly complain of phosphenes and dyschromatopsia. The optic disc can be swollen in one third of cases [6]. The Optic Neuritis Treatment Trial was a multicentre randomised trial that compared three treatment groups of patients with T-ON. The groups consisted of patients treated with intravenous corticosteroids followed by oral corticosteroids, oral corticosteroids only and a placebo group. Visual recovery was measured with a retroilluminated Snellen visual acuity chart and showed that most patients improved after treatment by at least 4 lines or more [14, 15].

Atypical optic neuritis (A-ON), unlike T-ON is more commonly bilateral, with more profound visual loss and it indicates an aetiology different from MS [6]. In A-ON pain can either be absent or severe. Non-recovery of visual acuity by 6 weeks, especially in non-white patients, is highly suggestive of A-ON [6]. A-ON is more common in areas where MS is less prevalent and in non-white populations [3]. A-ON can be caused by a variety of aetiologies including demyelinating, infectious and inflammatory diseases [3].

MS is not a common disease among the black South African population. A retrospective review by Bhigjee et al. in 2005, showed that the prevalence of MS in Kwa-Zulu Natal was highest for the white population (25.9 per 100 000) and lowest for the black population (0.22 per 100 000) [16]. A study by Modi et al. on the prevalence of MS in South Africa showed that most respondents in a survey were white patients [17].

MS-related ON and isolated ON with the potential of becoming clinical MS, generally accounts for T-ON. ON from other causes generally presents as A-ON. A-ON can be caused by auto-immune diseases such as Neuromyelitis Optica Spectrum Disorders (NMOSD) with Anti-Aquaporin-4 IgG antibodies (AQP-4 IgG), Myelin Oligodendrocyte Glycoprotein IgG antibody associated disorders (MOGAD); Systemic Lupus Erythematosus (SLE); Behcet’s disease or Granulomatosis with Polyangiitis (GPA) or systemic inflammatory diseases such as Sarcoidosis. These causes are important to identify early as they can lead to profound visual morbidity and require early detection and directed treatment to prevent severe visual loss. Infectious causes of ON include Tuberculosis (TB), Syphilis, Human Immunodeficiency Virus (HIV), Varicella Zoster Virus (VZV) and Bartonella Henselae (which usually causes a neuroretinitis) [3].

NMOSD has gone through significant advances both in diagnosis and treatment in the last 2 decades. The discovery of the anti-AQP4 IgG as a biomarker has meant that the disease is now known to be completely distinct and separate from MS, and therapies can be directed against these antibodies [18, 19]. NMOSD differs from MS in that the majority of NMOSD cases occur in African and Asian patients while MS predominantly occurs in Europeans [19]. NMOSD can have either a monophasic or relapsing course [18, 20]. In addition to ON, NMOSD has numerous other syndromes that it can present with and these are [21]:

■ Longitudinally Extensive Transverse Myelitis (LETM): motor, sensory, and continence abnormalities.
■ Area postrema syndrome: intractable hiccups or nausea/vomiting.
■ Diencephalic Syndrome: narcolepsy, hypersomnolence, hypothermia, hyponatraemia, behavioural changes and anorexia.
■ Acute Brainstem syndrome: oculomotor, motor, sensory and cerebellar dysfunction.
■ Cerebral Syndromes: hemisensory loss, hemiparesis, encephalopathy, cortical visual loss, and post chiasmal visual field loss.

These syndromes have characteristic Magnetic Resonance Imaging (MRI) findings which need to be present with the accompanying syndrome [21]. Magnetic resonance imaging is the imaging modality of choice when investigating the extent of optic nerve involvement, as well as to establish any features of the associated conditions. In the setting of MS, the involvement of the optic nerve is typically unilateral, short-segment, and confined to the nerve itself [22, 23]. In NMOSD and MOGAD, the involvement is more often bilateral and extends longitudinally into the optic chiasm and optic tracts [22–24]. Affected optic nerves demonstrate thickening and contrast enhancement. Enhancement of the optic nerve sheath and surrounding orbital fat may also be present in the acute setting [23].

These findings mirror the extent of involvement in the spinal cord with typically short segment (< 3 vertebral bodies) lesions in MS, and long segment lesions (LETM) in NMOSD and MOGAD. However, it is possible to encounter reversal of extent of involvement in these conditions [23, 24]. These findings manifest as increased signal intensity on T-2 weighted imaging (T2WI) and Short-tau inversion recovery (STIR) images, and may or may not inhomogeneously enhance with contrast administration. T-1 hypointensity may represent cord necrosis. Cord swelling may be noted acutely, while recurrent or intense long segment involvement may result in cord atrophy [24].

Additional brain imaging findings may be present. Anti-MOG lesions manifest in the brainstem, although a normal brain MRI is not uncommon [24]. In NMOSD, findings are often non-specific [23, 24]. However, lesions may be identified in the AQP4-rich periventricular and peri-aqueductal regions, as well as the area postrema and corticospinal tracts [22–24]. Hemispheric white matter involvement may be tumefactive, yet lack mass effect. The corpus callosum may be affected in both NMOSD and MS. While in MS, the lesions are discrete and extend perpendicular to the ventricles from the calloso-septal interface (Dawson’s fingers), the lesions in NMOSD follow the ependymal lining and are oedematous in the acute setting. Cortical, inferior temporal and S-shaped U-fibre lesions are also found to be specific and sensitive findings in MS. Brain manifestations are often T2WI and Fluid Attenuated Inversion Recovery (FLAIR) hyperintense. Enhancement may be patchy in NMOSD, while having an open-ring or well-defined configuration in MS [23].

Studies from Afro-Caribbean people in the West Indies have demonstrated that NMOSD has a predilection for patients of African ancestry [25, 26]. A meta-analysis by Musubire et al., also showed that NMOSD is more common in African patients, although all studies in this meta-analysis were retrospective [19]. This is extremely important in our country where the majority of the population is Black and where the exact prevalence of NMOSD is unknown. NMOSD can be fatal with a 5-year survival rate of 90% and 68% in those with patients with a monophasic and relapsing course respectively [20]. Early intervention and prevention of acute attacks in patients with NMOSD has been shown to improve visual outcomes [27, 28], with decreased retinal nerve fibre layer loss thought to be contributory [27, 29].

Early diagnosis and differentiation between MS and NMOSD is essential because treatments used for MS such as interferon beta, natalizumab, fingolimod, and alemtuzumab, may aggravate NMOSD [18]. MOG antibody-associated disorders may also present with ON and transverse myelitis (similar to NMOSD). In addition, features of acute disseminated encephalomyelitis (ADEM), seizures and even peripheral nervous system involvement may be encountered in MOGAD. However, unlike MS and NMOSD, MOGAD is more commonly a monophasic illness, thus chronic immunosuppressive treatment may be less frequently required [30–32].

To our knowledge there have been no large prospective cohort studies in a South African population on ON in terms of its clinical presentation, aetiology, treatment response and the effect or relevance of concomitant HIV infection.

## 2. Aim

The aim of this study is to describe the clinical characteristics, aetiology, response to treatment and clinical course of consecutive patients presenting with new onset ON (within one month) to Chris Hani Baragwanath Academic Hospital (CHBAH)/St John Eye Hospital (SJEH).

## 3.Objectives

**Primary objective**

- To determine the aetiology of ON in patients presenting with ON to SJEH/CHBAH and to assess the visual acuity outcome at one year post initial presentation.

**Secondary objectives**

- To describe the clinical response (including visual outcomes) to standard treatment in patients with ON and assess important clinical predictors on treatment outcome.
- To analyse the longitudinal course of the visual acuity recovery in these patients.
- To describe the demographic characteristics and relevant co-morbidities of consecutive patients presenting with ON to SJEH/CHBAH.
- To describe the clinical presentation and features of patients with ON presenting to SJEH/CHBAH.
- To describe any other neurological abnormalities associated with ON at presentation or subsequent follow-up.
- To describe the radiological characteristics on MRI of patients presenting with ON and correlate with clinical outcome.
- To identify and follow a subset of patients with NMOSD and MOGAD, assessing their clinical course and response to treatment.

## 4. Materials and methods

### 4.1 Design

The study will be a prospective, hospital-based cohort study. Patients will be followed up three-monthly over one year and the NMOSD and MOGAD patients will be followed up yearly thereafter for five years.

### 4.2 Study procedure

All consecutive adult (age 18 years and older) patients with ON presenting to either the neurology department at CHBAH or the ophthalmology department at St John Eye Hospital will be invited to participate in the study. St John Eye Hospital is the ophthalmology department forming part of Chris Hani Baragwanath Hospital, which is the largest public hospital in Africa. The patient demographic is mainly black African patients from low income households. Since this is not a study with two cohorts or differing interventions, no sample size can be calculated. We aim to enrol a sample of 100 patients. The following will be documented:

#### Patient demographics

Age, sex and self-reported race.

**Patient history**

- The patient’s main complaint including onset and duration of pain and decrease in visual acuity. Presence or absence of phosphenes and visual field defects. The presence of colour vision defects if noticed by the patient. The presence of any other neurological symptoms.
- Co-morbidities including smoking history and HIV status. If the HIV status is unknown the patient will be offered HIV testing. If the patient is HIV positive a CD4 lymphocyte count and HIV Viral load will be done. (HIV-positive patients not on treatment will be referred for anti-retroviral treatment).
- Past medical history including history of neurological symptoms such as paraesthesia, hemiparesis or hemiplegia, loss of bowel or bladder function, narcolepsy and hypersomnolence. Previous neurological assessment /referral will also be noted.
- Past ocular history including a previous history of optic neuritis, history of trauma, history of cataract surgery and prior treatment before presentation.

#### Clinical examination

The patient’s eye will be examined by the ophthalmology department (SJEH) for the following:

- Visual acuity: Corrected distance visual acuity (CDVA) using a Logarithm of Minimum Angle of Resolution (LogMAR) chart.
- Complete ocular examination including presence of a relative afferent pupillary defect (RAPD), optic disc swelling or macular star on the fundus.
- Colour vision testing using a Farnsworth-Munsell 100 hue test.

Neurological examination by the neurology department at CHBAH will include:

- Assessment for other cranial nerve palsies.
- Assessment of the patient’s tone, power, reflexes and sensation in both upper limb and lower limbs.
- Presence or absence of cerebellar signs.
- Presence of other syndromes indicative of NMOSD such as: Transverse Myelitis, Area Postrema Syndrome, Acute Diencephalic syndrome, cerebral and brainstem syndromes.

### Standard-of-care investigations

- Blood tests including full blood count and differential;; Syphilis serology; AQP-4 IgG and anti-MOG antibodies using immunofluorescence assays (Euroimmun Medizinische Labordiagnosstika AG, Lübeck Germany); serum angiotensin converting enzyme (sACE); Anti-Nuclear Antibody (ANA); and HIV testing and CD4 lymphocyte cell count (after consent for HIV testing is obtained).
- Lumbar puncture. The following tests will be performed on the patients’ cerebrospinal fluid (CSF): Biochemistry, microscopy culture and sensitivity (MC&S), Adenosine deaminase (ADA), Cryptococcal latex antigen (CLAT), India Ink stain, VDRL, Fluorescent Treponemal Antibody Absorption test (FTA-Abs), and viral panel PCR. Paired samples of CSF and serum will undergo electrophoresis with isoelectric focussing and IgG immunofixation in order to detect the presence of oligoclonal bands.
- Chest X-ray
- Visual field (VF) testing using a Humphrey visual field analyser (Zeiss, Germany) if the visual acuity permits it.
- Optical Coherence Tomography (OCT) and angiography will be performed using the Spectralis Spectral Domain OCT (Heidelberg Engineering, Germany). Scans will be performed of the peripapillary Retinal Nerve Fibre Layer (RNFL) as well as the macula. The OCTA scans will be performed on the optic nerve head and surrounding peripapillary retina as well as the macula.

Magnetic Resonance Imaging (MRI) of the brain and spinal cord within 72 hours of admission in order to identify any brain or spinal cord lesions in keeping with MS or NMOSD. Patients will be scanned on a Siemens Magnetom Aera 1.5T machine.

#### Sequences

*Brain*.

1. 3D- isotropic T1 (1mm)
2. 2D- axial T2 (5mm)
3. 2D- axial FLAIR (5mm)
4. 2D- sagittal FLAIR (5mm)
5. DWI (B1000) and ADC map
6. SWI

*Orbits*: (Orbits are included if current history of optic neuritis).

1. 2D- STIR- coronal (1mm)
2. 3D- isotropic T2 (axial 1mm)
3. 3D- T1 (fat- sat: axial and coronal 1mm)
4. 3D- T1 (fat-sat: axial and coronal mm) and 3D- isotropic T1 (1mm) of the brain post Gadolinium

*Cervical spine and (Thoracic spine if necessary)*.

1. 2D- T2- sagittal and (axials only if necessary)

Gadolinium is not routinely used.

f. Tuberculin skin test. In HIV positive patients an induration ≥5mm will be regarded as significant whilst in HIV-negative patients any induration ≥10mm will be regarded as significant.

Patients will be admitted for 5 days. They will receive intravenous corticosteroid treatment in the form of Methylprednisolone 1g per day for 3 days if admitted with Ophthalmology and for 5 days if admitted with Neurology. These are as per the normal treatment protocols. This may be followed by a tapering course of oral Prednisone according to clinical judgment of the treating team. Patients who do not respond or have incomplete recovery will be assessed and may receive plasma exchange at the discretion of the treating neurologists. Patients will then be followed up after admission at 1 month, 3 months, 6 months and every 3 months after that. They will be followed up for a period of 2 years. The NMOSD and MOGAD patients will be followed up annually for a period of 5 years.

### 4.3 Inclusion criteria

- All patients with new onset optic neuritis (unilateral or bilateral) presenting to the ophthalmology department (SJEH) or the neurology department (CHBAH).
- Patients over 18 years of age.

### 4.4 Exclusion criteria

- Patients with other optic neuropathies causing loss of vision such as non-arteritic anterior ischaemic optic neuropathy (NAION), glaucomatous optic neuropathy etc.
- Patients who do not give consent to participate in the study.
- Patients who are unable to undergo an MRI due to a metallic prosthesis or implant.

#### Materials, equipment and facilities

The Spectralis SD-OCT (Heidelberg engineering, Germany) available at SJEH will be used.

The Humphrey Visual Field analyser (Zeiss, Germany) will be used to assess patient’s visual fields.

Visual acuity (VA) charts and phoropters for refraction are available in the ophthalmology department.

MRI scanning on a Siemens Magnetom Aera 1.5T machine.

### 4.5 Data collection

Information collected will contain the following:

1. Personal information: Patient Code, Age, sex, HIV status, other medical co-morbidities, symptoms including loss of vision, presence of pain, phosphenes, Lhermitte’s phenomenon, Uhthoff’s phenomenon.
2. CDVA at baseline, day 4 or 6, 1 month, 3 months, 6 months, 9 months and 1 year. The patients with NMOSD will be followed up annually for 5 years. LogMAR VA will be recorded.
3. Details of ocular examination including visual acuity, pupil abnormalities, optic disc swelling, colour, brightness and contrast sensitivity.
4. Results of neurological examination.
5. Colour vision testing, visual field and OCT results.
6. Blood and cerebrospinal fluid (CSF) results.
7. CXR and tuberculin skin test (TST) results.
8. MRI scan results.
9. Treatment and duration of treatment given.

### 4.6 Data management and statistics

Patients’ data will be entered and stored on the REDCap software (Vanderbilt University, USA) [33, 34]. Data will be analysed using the statistical package STATA (version 17). Statistical significance will be set at 0.05. If multiple tests are used and there is a need for correction of the family-wise error rate, this will be factored in at the analysis stage.

Baseline continuous variables will be analysed with the t-test if they are normally distributed or with the Wilcoxon rank sum test if they are skewed.

The Pearson Chi-square test will be used to analyse the differences in demographics / clinical signs (gender, ocular pain, bilateral involvement, recurrence and papillitis) between the different groups of patients.

The primary outcome describing the aetiology of optic neuritis in our population will be analysed using proportions/percentages. Univariate/Multivariate regression analyses will be employed to assess if there are clinical predictors such as aetiology for good visual and clinical outcome. Multilevel models will be used to assess the visual acuity over time.

### 4.7 Time schedule

The study is anticipated to commence in May 2022. It is anticipated that it will require two years for enrolment of patients to be completed. Patients will be followed up for 1 year, unless they are diagnosed with NMOSD or MOG optic neuritis. This subgroup from the original cohort will be followed up for 5 years.

### 4.8 Ethical considerations

#### Human Research Ethics Committee (HREC) approval

The study has been approved by the University of the Witwatersrand Human Research Ethics Committee (HREC) study number M180764, and the study will be conducted in accordance with the tenets of the Declaration of Helsinki. No names, hospital numbers or any other information that can breach patient confidentiality will be published.

Interpreters/translators will be available should they be required, to explain to patients the purpose and risks of this study.

*Risks*. Patients can feel some discomfort during the drawing of blood and the lumbar puncture. These are, however, standard care for the condition. Patients may experience hyperglycaemia during standard-of-care treatment with intravenous steroids. Patients will be managed appropriately by doctors if the patient develops any adverse or side effects as a result of the procedures or treatment.

*Confidentiality*. Each patient’s hospital number will be assigned a unique code. This code will appear on the data collection sheet together with the information obtained from the patient’s file. The file linking the patient code to the patient hospital number will be kept separate from the datasheet and will only be used for crosschecking purposes. Strict confidentiality will always be maintained.

## 5. Discussion

This study is the first large hospital based prospective study to be conducted on ON in Southern Africa. African populations do not usually present with T-ON^6^ and no large studies have been conducted in Sub-Saharan Africa to ascertain the presentation, aetiology and outcomes of A-ON. This is a first important step to understanding the presentation and outcomes of patients presenting with ON in an African context.

The hospital-based nature and geographic location may result in decreased generalisability to other regions in Southern Africa, as well as the rest of Africa. The study will be published in a peer-reviewed academic journal once it is completed and analysed.
